# Forensic interviews conducted with autistic adults in Japan: a review of the literature and directions for future research

**DOI:** 10.1080/13218719.2023.2192255

**Published:** 2023-04-26

**Authors:** Dave Walsh, Graham Brooks, Makiko Naka, Gavin Oxburgh, Akira Kyo

**Affiliations:** a School of Law, De Montfort University, Leicester, UK; b School of Human and Social Sciences, University of West London, London, UK; c Department of Psychology, Ritsumeikin University, Osaka, Japan; d Department of Social Sciences, Northumbria University, UK; e Department of Law, Kwansei Gakuin University, Nishinomiya, Japan

**Keywords:** autism, developmental disorders, Japan, police, police interviewing, public prosecutors, suspects, victims, vulnerabilities, witnesses

## Abstract

The interviewing of victims, witnesses and suspects is important in helping resolve criminal investigations. In Japan, developments have recently occurred in the training of the police and their public prosecutors in these key tasks. Whilst literature exists on autism in Japan, studies examining police/public prosecutor interviews with autistic adults conducted in that country (and indeed, any other) remain scant. As elsewhere in the world, identification of those who manifest characteristics prevalent on the autism spectrum disorder (ASD) scale, has been found to be problematical to criminal justice professionals. To help address this deficit in understanding, we provide an overview of the literature concerning contemporary understanding of the challenges facing autistic adults as they attempt to reveal their verbal accounts, as well as suggested techniques when interviewing adults on the ASD scale during criminal investigations, offering lessons learned from research conducted around the world that provide potentially promising solutions for Japan.

## Introduction

Interviews with victims, witnesses or suspects of crime have been cited as one of the most important aspects of resolving criminal investigations, as well as one of the core investigative tasks of police officers that is most frequently undertaken (Oxburgh & Ost, 2011; Walsh & Bull, 2010). Studies conducted around the world over the last thirty years have proliferated concerning the undertaking of such interviews. Indeed, particularly over the last 10–15 years the amount of such research conducted in Japan has also grown. However, there remains a dearth of research relating to police interviews with those who are autistic (a disorder that is believed to affect around 3% of the Japanese population, Sasayama et al., 2021). What research has been conducted in Japan concerning vulnerable interviewees has largely tended to focus on children (Naka, 2016). In contrast, in this paper, we review the literature conducted around the world specifically in the context of those adult interviewees who suffer from autism spectrum disorder (ASD) who have previously been found to present particular challenges for criminal justice professionals (Maras et al., 2020).

This paper discusses many of these, and includes recent research that has sought to mitigate and overcome them. From this literature base, we examine the context of criminal justice professionals (e.g. the police and Public Prosecutors) in Japan. Public Prosecutors from that country have consistently discussed at various international conferences and symposia during the last 3–4 years (at which the authors have been present) how they feel insufficiently equipped to either identify or interview such individuals (a key task in their job role). Those discussions yielded suggestions that they also consider that the Japanese police are similarly challenged. As such, this paper intends to provide a platform for training developments for these professionals.

## The Japanese criminal justice system and ASD

Developments occurred in Japan in 2012, when the National Police Academy (NPA) launched a programme to advance investigative interviewing methodology and published a basic training manual for such interviewing that includes information on the psychology of memory, false confessions, questioning styles and how to avoid leading questions (Wachi & Watanabe, 2016). The manual was developed in collaboration with psychologists and draws on approaches used in Western nations, such as the PEACE information-gathering model from England and Wales (Shepherd & Griffiths, 2021). In 2013, the NPA established the Research and Training Center for Investigative Interview and Interrogation Techniques to deliver training in this area. Such reform reflects the miscarriages of justice that occurred in 1990–2003, where police interviews were thought to elicit false confessions (NPA, 2010; Supreme Prosecutors Office, 2009). In the case report, the suspect was described to have the characteristics that were ‘introversive and unsociable, and not able to refute if talked strongly’ (NPA, 2010, p.5). It is also noted that intellectual ability of suspects should be considered when conducting investigative interviews (NPA, 2010, p.19). Thus, at least there seems to be an awareness to the importance of the attributes of suspects.

With regard to the training and educational development of public prosecutors, reform has been also made over the last 10 years, which is thought to have been prompted by widespread concerns as to the need for increasing the skills required by criminal justice professionals (Ramseyer & Rasmusen, 2013; Saegusa, 2009; Watson, 2016). Public prosecutors now receive a range of academic tuition and legal training, plus several months practical experience working with actual cases ‘in the field’, such as in the courts, and public prosecutors’ offices intended to develop skills when dealing with victims, witnesses and suspects of crime (National Legal and Training Research Institute, 2021). Still, it remains doubtful whether the police and public prosecutors feel sufficiently able to identify and deal with interviewees’ vulnerabilities. Recent work undertaken by the authors over three years suggests that such professionals are seeking to develop their existing skills to undertake their duties.

Wachi et al. (2014) conducted a study of 276 Japanese police officers, finding that they stated a preference for information-gathering strategies. However, despite interviews with suspects being electronically recorded since 2019, there has still been no study that has observed how they actually conduct these interviews. As such, despite the reform, what strategies (whether information-gathering or confession-seeking) are undertaken in practice remains unknown. Shigematsu (2013) asserted that confession-oriented methods still prevail in Japan. At the same time, Wachi et al. (2016) found that some convicted offenders reporting experiencing these particular techniques when undergoing police interrogations. Nevertheless, in that study, others said that their interviews were characterised by rapport-building approaches, which had encouraged them to disclose information. It is, thus, necessary to follow up the study of interviewer perceptions by analysing the actual video-recorded conversation between the interviewer and the suspect in order to establish what occurs in these interviews.

Although there has been little research in Japan concerning interviews with autistic individuals, a small-scale study (see Watanabe et al., 2016) was conducted with 21 police officers who were asked how they approached interviews with those interviewees possessing intellectual disabilities (ID). While that category of individuals ought not to be conflated with autistic interviewees (although 30% of autistic people are reported as also having an ID; Baio et al., 2018), Wachi and Watanabe (2016) found that police officers said they tended to make adjustments for those interviewees they classified as having an ID. For example, they said they explained to interviewees that they should not guess an answer if they did not know. Police officers in that study also advised that building rapport was important. These researchers also found that the police officers were generally satisfied in the amount of information they obtained from interviewees. However, they also admitted that they did find it demanding to make themselves understood when questioning such individuals.

## Police officers identifying and responding effectively to autism

Regardless of which interview techniques are effective in gaining maximum information from interviewees, they are all dependent on whether police officers are able to recognise when their interviewee has autism. Farrugia (2022) advises that police officers around the world (including Japan) do not receive sufficient or specific training to be able to effectively identify interviewees who are potentially vulnerable due to being autistic (or indeed for that matter, those with other vulnerabilities such as their age or their having intellectual disabilities). Alleley (2015) has also stated that those with autism present even further problems concerning their identification by police officers, since those possessing high functioning ASD are often very intelligent and linguistically competent, if occasionally somewhat unorthodox in their use of language (Murphy, 2018). However, such presentational behaviours can conceal their potential vulnerability (I. Freckelton, 2007). Unsurprisingly, therefore, previous research has found that police officers often experience difficulties in interviewing these individuals (Dehagani, 2019; Geijsen, 2018; Gudjonsson, 2003; McKinnon & Grubin, 2013; Young et al., 2013). In addition, Mergaerts (2021) reports that, despite the identification of vulnerability, responses to any recognition by the police are not always adequate ones that safeguard such individuals – and their testimony (also Dehagani, 2019; Farrugia & Gabbert, 2019; Geijsen et al., 2018; Gudjonsson, 2003, 2018). For example, Vaughan et al. (2019) found that the police in England and Wales stated that they duly considered expert advice when considering how they should interview vulnerable suspects. However, these authors also found that the police did not consider asking for further opinion on these interviewees while they conducted ongoing interviews, nor did they ask for further assessment once these various interviews had concluded. That is, any increased vulnerability associated with custodial incarceration was not considered.

Recent data have revealed that rates of ASD in Japan hover around 3% of the population (Sasayama et al., 2021). How such individuals are identified, viewed and treated by law enforcement and public prosecutors in Japan remains under-researched, although the Japanese National Research Institute of Police Science (NRIPS) has developed N2-FAST, a brief screening tool that can be used to identify interviewees with intellectual disabilities (see Watanabe et al., 2016). Furthermore, in Japan, the gathering of information from victims, witnesses and suspects is mainly the responsibility of the police, prior to them passing the case on to the Public Prosecutors’ office. While learning disorders are usually determined prior to interview by the police or prosecutors, through IQ tests, developmental ones (such as autism) are inadequately assessed. Although around 30% of autistic individuals may have an associated learning disability, specific challenges remain insufficiently understood concerning the interviewing of autistic adults (and, in turn for those interviewees to relay verbal accounts). It also falls solely to Public Prosecutors to evaluate the credibility of the testimony of suspected offenders, victims and witnesses in order to decide whether or not to prosecute the case. Public Prosecutors can also request an appraisal or assessment if they suspect, after the interview, that the interviewee has ASD. This, as with other jurisdictions though, can be sometimes too late and can lead to a failure on the part of the police and prosecutors in Japan to incorrectly recognise someone with ASD. That is, the consequences of identification at this point in the investigation could be the failure to treat an interviewee’s account seriously or appropriately.

Given the limited guidance they receive, autism might be misdiagnosed as, for example, simply a matter of obtuseness or awkwardness in an interview (Crane et al., 2016). Worse still, individuals with autism, however (as we have noted), do not always openly declare their status or are capable or willing to offer such a declaration (Crane et al., 2016). As such, as in other parts of the world, individuals with ASD can be incorrectly diagnosed during the criminal justice process (Alleley, 2015). A greater understanding of such matters would be invaluable to investigators when preparing for an interview with victims, witnesses and suspects (Mandy et al., 2018).

## Autism and the criminal justice system

Those with ASD are more than likely to live law-abiding lives (Murphy, 2018). Research suggests, still, that those who are affected by ASD (particularly those who display behaviours that might be viewed as different) possess a higher chance of coming into contact (for whatever reason) with the police than non-autistic people (Baldry et al., 2012; Debbaudt, 2002; Debbaudt & Rothman, 2001). Moreover, once in the criminal justice system (CJS), autistic individuals react in many different ways (see Brewer & Young, 2015; Siponmaa et al., 2001) that are directly associated with their autism (Allely & Cooper, 2017). However, it has been found that some police officers often fail to identify those with autism, misinterpret their behaviours, and thus undertake incorrect tactics themselves when engaging with this particular population (i.e. when they are interviewed as a victim, witness or a suspect of crime; Maras et al., 2020). Such increased incidence of involvement in the CJS for those with ASD may be related to their different vulnerabilities since they may well possess some difficulties in their thinking, reasoning and judgements, as well as increased challenges in communicating effectively, understanding others and to be understood themselves (Aldridge et al., 2000). They may also be characterised by problems in relation to their education, employment, contact with the police and the wider CJS, personal responsibility or independence, all associated with an increased lack of understanding regarding risk and the consequences of any risk-taking behaviours (Maras & Bowler, 2014).

Young and Brewer (2020) commented that increased engagement with the police relates to a ‘theory of mind’ standpoint. That is, those with autism, once suspected of criminal involvement (where those suspicions were, in fact, inaccurate ones), would tend to have greater difficulties than non-autistic adults in both explaining their innocence and removing themselves from those suspicions, as discussed in more detail in the following section. Young and Brewer also add that autistic people may have difficulty with taking the perspective of police officers, which can lead to their neglecting to judge whether their explanations were satisfactory or understood.

The research conducted so far on suspected offenders with ASD, however, has failed to deliver cogent evidence in supporting clear established links between autism and crime (National Autistic Society, 2017). Prior research is undoubtedly useful, but often limited since samples used are mostly on suspected or known offenders (and thus excluding those yet to be known, suspected or convicted). Research has also been problematised by a limited number of cases that fail to isolate the effect of autism where there is co-morbidity with other mental health, intellectual or learning disorders, which has been argued as explaining any disproportionate levels of offending in the autistic population (Alleley, 2015).

### How does ASD present?

Autism is a neuro-developmental disability; however, it is neither an illness nor a learning disability (although some of those on the ASD scale may also have a mental health issue or learning disability). As a lifelong condition with underlying neurological causation, autism may be better described as a developmental disorder with socialisation and communication issues, presenting in diverse ways across a spectrum (Chown, 2010). ASD can present with co-morbid conditions (e.g. anxiety, some mental health conditions, substance abuse etc.), personality disorders or socio-demographic conditions that can contribute to its manifestation in a mild or serious form (Casanova et al., 2020, see Murphy, 2018, for a more detailed overview of ASD).

For anyone, encounters with law enforcement can be a stressful experience, but some of the features of autism exacerbate such experiences for those on the ASD scale (Alleley, 2015). Their challenges with communication skills could cause them to fail to understand criminal justice processes and procedures. At the same time (and with particular relevance to the focus of this paper), some autistic people recall their own memories for events by employing what might be viewed by police officers as either a very formal or quite unorthodox approach to storytelling. There may, for example, be a tendency for them to engage in extended monologues or they may fail to understand figurative language (Brewer & Young, 2015; I. Freckelton & List, 2009). Besides, autistic individuals are reported to have difficulties, when recounting from memory, either recalling the chronological sequence of events or those personally experienced ones (Bigham et al., 2010). Such difficulties can potentially cause problems for those on the ASD scale in giving testimony, whether pre-trial (i.e. in police interviews) or later in court. Despite this concern, the police and prosecutors in the CJS are expected to be aware of a diverse range of symptoms that those with ASD can present (Crane et al., 2016, Hepworth, 2017; Snook et al., 2012). However, if these professionals have not been sufficiently trained to understand ASD, there is a significant risk that interviewees’ behaviour may be misattributed or their testimony misinterpreted (Crane et al., 2016). Moreover, some autistic people may not always (or immediately) be identified as such by the police or Public Prosecutors. As Alleley (2022, p.23) advises, ‘* . . .* one of the primary reasons for this may be their apparent competent use of language and the fact that they appear to be intellectually capable*’*.

A lack of awareness and knowledge by criminal justice professionals of the range of conditions can adversely impact criminal justice cases, potentially leading to miscarriages of justice (Beetham, 1991; Bottoms & Tankebe, 2012; Brooks, 2019; Tsushima & Hamai, 2015; Tyler, 2003; Tyler & Wakslak, 2004). Since ASD is characterised by difficulties in accurately assessing and understanding social situations and/or other people, there is an increased likelihood of their coming into contact with the police, whether as a victim, witness or suspect (Brown-Lavoie et al., 2014; Chaplin & Mukhopadhyay, 2018; Heeramun et al., 2017; Tint et al., 2017; Weiss & Fardella, 2018). Such worldwide research has brought greater awareness concerning how people with autism might behave in certain stressful situations (such as being involved with the CJS). In particular, those with ASD may face particular demands concerning their effective communication with those undertaking law enforcement, such as the police (Murphy, 2018). It should not be surprising that researchers have increasingly turned their attention to encounters between these criminal justice professionals and those affected with autism, including when they are interviewed as part of a criminal investigation.

## Police interviews/interrogations with autistic people

Interviews/interrogations are a process of social interaction and communications. As has been noted, those conducted by criminal justice professionals (such as the police and Public Prosecutors) during the course of their duties may present particular challenges to those with autism. Their responses to these situations can, in turn, present difficulties for these professionals, especially those who do not have sufficient skills, knowledge or training to deal with autistic people. Regardless as to whether the interviewee with autism is a victim, witness or suspect, the experience of being interviewed by the police is highly likely to be viewed or experienced as a stressful encounter (North et al., 2008). In such a situation, those interviewees may struggle to cope with the demands placed upon them by the police, even if they are seeking information, rather than a confession to a crime. The autistic person is then likely to exhibit signs of distress. Alleley (2015) reports that such demonstrations may be subject to negative interpretations by interviewing officers, regardless of whether they are a victim, witness or suspect. For example, signs of anxiety may be viewed as signs of deceit, a common misconception (see Vrij, 2008). Of course, experiencing stress during police interviews will also apply to non-autistic people.

A technique argued as related to lessening interviewees’ anxieties in these situations concerns rapport-building, known to be associated with obtaining increased levels of information (Abbe & Brandon, 2014; Gabbert et al., 2021; Walsh & Bull, 2012). As such, alongside good questioning techniques and strategies, the development of rapport is a key feature of those interviews hallmarked by their approach to the gathering of reliable and detailed information. However, in the cases of those with autism, rapport building has again been found to possess extra demands. For example, an experimental study conducted by Crompton et al. (2020; though based in a neutral, non-forensic, domain) found that information communicated between autistic and non-autistic people was of an inferior quality when compared to those in other conditions. Moreover, lower levels of rapport were disclosed by those individuals in the mixed group too, possibly associated with autistic interviewees displaying suspicion or mistrust towards their interviewers (Blackshaw et al., 2001). Such behaviours, emerging in a criminal investigation, might result in the police viewing them as highly uncooperative, confrontational or even defiant (Alleley, 2015; Murrie et al., 2002; North et al., 2008) and, thus, perceived as having something to conceal (Walsh & Milne, 2007). At the same time, Gudjonsson (2003) advises that certain autistic individuals who suffer from certain conditions (e.g. paranoia, anxiety or depression) are: (a) overly compliant; (b) eager to please authority figures (such as the police and Public Prosecutors); and, as such, (c) are more willing to make self-incriminating statements, which may well include false ones.

Other possible behaviours, less associated with compliant interviewees, have been enlisted as those that autistic people may demonstrate under such interview conditions. These include these interviewees often requiring much longer to both process questions and provide answers (Crane et al., 2012), whilst (without interviewer guidance) failing to appreciate reciprocation of turn-taking roles during conversations with interviewers (Murphy, 2018). They may also possess difficulties in dealing with more open questions (White et al., 2009) or may need specific prompts to help with their recall (Maras et al., 2013). Other behaviours exhibited by autistic interviewees may also consist of speaking in monotone, expressing themselves either bluntly or very literally (also failing to understand either metaphors or subtle messaging), while seemingly lacking emotion (Murphy, 2018). They may also:

* . . .* have an impaired or no ability to speak; lack of eye contact; an insistence on sameness; an attachment to objects which is considered obsessive; self-stimulating behaviour such as hand flapping, body rocking, or attachment to objects; inappropriate behaviour (for instance, laughing during a serious situation such as a police interview or during court proceedings); no sense of fear in response to danger; hypo- or hyper sensitivity in response to pain; tantrums (often referred to as ‘meltdowns’) or escalated behaviour for no apparent reason and a preference to be alone. (No author, *AELE Law Journal*, 2009, cited in Alleley, 2015, p.2)

Recommendation of a series of preventative and countermeasures to be undertaken by the police in dealing with such matters and help building of rapport include: (a) maintaining a safe distance; (b) avoiding invading their space; (c) modelling behaviours as an example for them to emulate; (d) having a calm disposition and voice; (e) using simple language; (f) avoiding touching, and (g) accommodating a delayed response to questions and instructions (Alleley, 2015). However, what remains insufficiently examined in research is whether such responses do indeed build successful rapport with autistic individuals, and if (after any rapport has been built) there is an associated increase in information quality and quantity.

In addition, Al-Attar (2018) focused on terrorist suspects who were highly functioning autistic individuals. These suspects exhibited more sophisticated behaviours (manifested, say, in enhanced language skills) that might mislead police officers to judge that their suspects possess highly developed social, communication and emotional skills, whilst overlooking their autism. Moreover, Al-Attar contends that such estimations of their suspects may lead police officers to view typical autism characteristics (e.g. repetitive and unusual behaviours, monotone lengthy and detailed statements, replete with candour) as signals of their possessing strong ideological dogma and commitment.

Al-Attar (2018) identified seven facets of autism that, she argues, need to be considered by interviewers in order for them to formulate the appropriate interviewing strategies, make the necessary adjustments and adapt their own responses during interviews. They relate to, in summary, autistic interviewees possessing: (a) an intense focus on circumscribed interests; (b) a rich vivid fantasy, though impaired, social imagination; (c) a requirement for a set orderly regime governed by rules and rituals and thus containing predictability; (d) obsessiveness, manifest in repetitive behaviours and collection of items, and so on, relating their intense focus or interest; (e) social and interaction difficulties; (f) particular cognitive functioning (that can range from impaired to sophisticated), and (g) sensory processing. Such articulation of these facets has led Al-Attar to make 20 recommendations that are contended to underpin effective approaches to interviewing (see Al-Attar, 2018, pp. 334–335). While Al-Attar describes these recommendations as empirically-based ones in their construction, what remains to be known is whether they possess efficacy in practice.

Murphy (2018) provides further guidance that includes interviewers making enquiries of relatives, social workers and other associates to help develop a profile that informs how particular interviewees respond when communicating. For example, these enquiries might well prompt interviewers to ask before the interview commences of an individual (who is known to possess particular sensitivities to noise or light) whether any environmental factors present might be distracting or causing irritation. Furthermore, Murphy advises that for those interviewees known to dislike close proximity to others, seating arrangements should be organised in the interview room in order to accommodate such particular problems.

### Specific approaches in suspect interviews

With further regard to those who are interviewed/interrogated as suspects of crime, there are two contrasting approaches that dominate police techniques across the world, regardless of the suspect’s vulnerability. The first approach is typified by police officers seeking confessions from suspects who are presumed as guilty, whereas the second focuses on the gathering of reliable and comprehensive information and open-mindedness as to their involvement in crime (Walsh et al., 2016). As we have already noted, Wachi et al. (2014) found that Japanese police officers preferred a rapport-based approach that has been found particularly prevalent in information-gathering interviews (Walsh & Bull, 2012). However, since there has been (as far as we know) no observational study of their contemporary practice, we are unaware which of the two methods they undertake when interviewing suspects. That said, anecdotal evidence suggests that a confession-based approach may exist in Japan that has been argued to be associated with conservative decision-making by public prosecutors as to whether to proceed with prosecutions unless evidence is incontrovertible or the suspect has admitted their involvement in wrong-doing where the evidence is less persuasive (Walsh, 2020).

Those interviews where confession-seeking is viewed as the police’s main aim when interviewing/interrogating suspects may well cause those with autism added difficulties. That is, interrogators in such contexts use a range of techniques to prompt confessions such as: (a) minimisation (where, briefly, the intent is to lessen the seriousness of the matter); (b) maximisation (where the intent is to show to interviewees that the consequences of continued denials is worse than making immediate confessions); (c) providing rationales for the commission of the offences (in order to provide ‘understandable ‘excuses), and (d) providing situational futility (where interviewees are told that it is pointless to deny offending as they will not be believed). These interrogation techniques have been found to be associated with false confessions (Gudjonsson, 2018), particularly the case with vulnerable interviewees. These interrogation strategies also fail to accommodate increased levels of compliance with people of authority that, for example, those with autism are known to demonstrate (Maras et al., 2020; North et al., 2008).

Moreover, certain police interview/interrogation techniques themselves may lead to autistic, and indeed, non-autistic, people (e.g. when denying their involvement in crime) in being incorrectly identified as liars. That is, police officers may consider that certain behavioural responses to questions that emerge (e.g. gaze aversion, response latency, fidgeting, etc.) are signs of deception, even though these behaviours have been found to be unreliable channels of proof of lies or truth (Vrij et al., 2019). Indeed, gaze aversion, for example, is quite commonplace in ASD individuals, regardless of whether they are telling lies or the truth. Thus, it would be incorrect to attribute that response as an indicator of deceit. If such responses are accompanied by ASD suspects denying their involvement (or unwittingly providing false information, due to their misunderstandings) the concern is that the police may believe that they are lying through either failing to identify interviewees’ autism, or misinterpreting behavioural responses (Luke, 2019; Strömwall & Granhag, 2003). Furthermore, rather than deliberately misleading the police, Talwar et al. (2012) argue that autistic interviewees can misunderstand police questions (due to their limited ‘theory of mind’), and so unwittingly provide false information and thus appear deceitful.

Several studies have also found that unexpected and atypical responses by autistic people (such as recurring and unusual body movements, gaze aversion or sustained gazing) led to increased presumptions of guilt (Lim et al., 2022; Logos et al., 2021; Maras, Crane, et al. 2019; Maras, Marshall, et al., 2019). However, these studies have also found that once the individual was identified as being autistic, the levels of guilt presumption and deception abated (see also Berryessa et al., 2015). These findings do suggest that identification of autism is a key factor, yet, Modell and Mak (2008) found that many police officers struggled with identifying ASD (also Chown, 2010). Additionally, Crane et al. (2016) found that people were frequently reluctant to willingly disclose their autism. This was not just due to them being unaware of their own autism, but also due to matters such as not seeing the condition as relevant, inability to disclose, or fear that disclosure would either lead to police victimisation or their evidence being disregarded.

In contrast to interrogations, with a central aim of obtaining confessions, police interviews with either victims, witnesses or suspects of crime are viewed as primarily an important means of gathering information to gain detailed and reliable accounts from them (Walsh & Bull, 2010). That is, all interviewees are fundamentally treated as witnesses to the incident(s) under investigation. This principle even includes suspects, where officers are trained to preserve an open mind concerning their apparent guilt to allow for other possible explanations that might indicate their non-involvement in any offence. As such, it is important that even they are given an opportunity to provide a comprehensive account.

The gathering of detailed accounts necessarily entails asking people to recall from memory (and occasionally requiring them to remember events or faces from several years earlier). However, autistic people have been found to face particular challenges in meeting such requests. An extensive body of research has found that those with autism have difficulties recalling and then freely providing accurate, organised, coherent and comprehensive accounts of episodes thateither they possess knowledge of or have themselves experienced (Adler et al., 2010; Almeida et al., 2019; Barnes and Baron-Cohen, 2012; Bowler et al., 2008; Chaput et al., 2013; Cooper et al., 2016; Crane et al., 2012; Gaigg & Bowler, 2018; Henry et al., 2017; Maras & Bowler, 2010, 2012; Mattison et al., 2018, Tanweer et al., 2010). Besides, having problems with providing a clear and unambiguous story, research studies have also found that autistic people insufficiently consider, if at all, whether they are making themselves understood through organised and well-scaffolded memorial recall as effectively as non-autistic people can (Baron-Cohen, 1988; Colle et al., 2008; Goldman, 2008; Hilvert Davidson & Gámez, 2016; Tager-Flusberg et al., 2005).

In response to such challenges, an alternative approach to police interviewing may be to ask more closed questions in gathering an account, yet such methods are known, in many cases, to provide further complications for criminal investigations and, indeed, for criminal justice per se. For example, there is a wealth of research that strongly suggests that closed questions are inappropriate and largely dependent on what the interviewer knows and believes as significant (but such awareness may be inaccurate and is likely incomplete). In any event, questions that are closed (or otherwise deemed inappropriate) will probably obtain only fragments of accounts and not the fuller details that are required (Oxburgh et al., 2010). However, whilst research advocates for the use of best practice interviewing methods (such as the use of open questions), there is an emerging branch of research that casts doubt on the appropriateness of open questions and the free recall technique for vulnerable populations. For example, more recently, Farrugia and Gabbert (2019) found that the use of open (or appropriate) questions may not be suitable for suspects with mental health conditions and disorders. As previously noted, autistic people often have such additional conditions.

### Use of the cognitive interview with autistic adults

The Cognitive Interview (CI) has regularly been found to yield more information from interviewees (Fisher & Geiselman, 1992; Memon et al., 2010). In brief, the CI involves a set of interviewer instructions to the interviewee, grounded in scientific research, such as that relating to the way memories are first encoded (Tulving & Thomson, 1973) and how through differing pathways they can be later retrieved (Wickens, 1970, and see Geiselman et al., 1984, for a fuller description of the CI). However, the CI has also been found problematic when it was used as a framework to interview those with autism (Maras & Bowler, 2010; Maras et al., 2014). That is, those with autism performed no better in recalling accurate details than those whose interviewers used a standard set of instructions to interviewees for them to tell everything that they saw. This may well be due to the greater concentration required of interviewees in the CI protocol. Adaptations have been offered to overcome such matters. For example, Mattison et al. (2015, 2018), and also Maras et al. (2014) found that autistic interviewees, when asked to draw a sketch of the context re-instatement details, provided more accurate details than those on the ASD scale, who undertook the traditional approach of narrating them. However, they reported fewer details than their non-autistic counterparts, and also those details contained more inaccuracies.

By continuing to use extant good practice interview techniques (i.e. the use of open questions) with this population, such strategies may well lead to autistic interviewees providing accounts they feel will meet interviewer expectations. However, such beliefs again can lead to either (or both) inaccurate and incomplete accounts being gathered (Fisher & Geiselman, 1992). That is, they are not as equally resistant to suggestible questions as non-autistic people (Maras & Bowler, 2012; North et al., 2008). At the same time, autistic individuals can also be more malleable and (if they feel under pressure to give information) have been found more likely to provide conjecture, rather than feeling able to declare that they cannot recall (or just do not know) something (Chandler et al., 2019; North et al., 2008).

#### Witness aimed first accounts

Recently, Maras et al. (2020) have developed an alternative approach called the Witness Aimed First Account (WAFA). Essentially, this approach involves facilitating witnesses, in the first instance, to provide their own free narrative by asking them what they attributed as most important about the event (which was then recorded by the interviewer on sticky repositionable notes that were then placed on the wall in the interview room). After being advised that they would return to this matter later, they were then asked to tell the interviewer something else about the matter that had happened. Again, their statement was manually noted, as before. This procedure was repeated until no further details could be provided. At that point, the interviewer returned to the manual notes (in the order originally given), and interviewees were then asked to give a free narrative about that particular aspect of the event, followed by probing questions to gain that further detail. ASD participants were duly found to provide more (and more accurate) information than in the CI condition or by asking for a free narrative from ASD interviewees. The initial segmenting of the story in the WAFA seems to be beneficial therefore. However, more testing is needed, particularly as the researchers acknowledged that they did not examine whether the story provided by their interviewees appeared as a coherent one (but just measured for the number of details provided). Narrative coherence is important to influencing others’ perceptions as to the credibility of the given account (Crane et al., 2018). Notwithstanding these matters of concern, the research of Maras et al. (2020) does provide a possible way forward, through supporting those on the ASD scale to give an accurate account that does not compromise its integrity or the criminal justice process.

## Conclusion

Concerns remain as to the quality of police training in identifying and interviewing appropriately those with such spectrum conditions (Adebowale, 2013; Chown, 2010; Crane et al., 2016; Dando et al., 2008; Hepworth, 2017; Maras & Bowler, 2010, 2014; Medford et al., 2003; Ministry of Justice, 2011; National Autistic Society, 2017). Allely and Cooper (2017) have stressed the importance of criminal justice professionals being provided with suitable and specialised training to undertake such interviews. What literature exists is mostly devoted to criminal justice practice with emphasis upon suspected offenders, but less so witnesses and victims with ASD (Crane et al., 2015).

For child victims, recent developments have occurred in Japan, with the introduction of ‘cooperative interviews/representative interviews’. That is, the public prosecutor, law enforcement and social workers from child guidance centres conduct together a forensic interview, based on such protocols as the NICHD[A9] investigative interviewing protocol (for more on this protocol see La Rooy et al., 2015). Moreover, since April 2021, in 13 major prefectures in Japan, these representative interviews have also been conducted for adult victims identified with mental disorders and those described as ‘intellectually challenged’ people (Hope et al., 2022). If such a multi-disciplinary team (MDT) approach is taken, social workers, medical staff or other professionals in the team may identify the ASD or other difficulties in the interviewee, and support the interview. For instance, in one case in Japan, a prosecutor interviewed a child victim who was suspected to have ADHD by a school teacher of the child (Yamada, 2021). The prosecutor then asked for assistance from the school teacher and social workers at the child guidance centre in planning and conducting the interview. Those with ASD may, however, belong to neither one nor the other of these two groups, nor is an MDT approach usually undertaken for interviews with suspects.

Despite other developments in training, it remains the case that, whether victims, witnesses or suspects, such determinations of vulnerability are expected to be recognised by the police and public prosecutors. Yet we strongly suspect that the police and Public Prosecutors are likely to have at best only a partial understanding as to how to arrive at such recognitions of those with autism. If a suspect fails to disclose their autism to the police (as we have seen is often the case), who themselves may lack understanding of ASD, it falls to Public Prosecutors to identify any affected individuals. In scoping information for this paper, we learned that Public Prosecutors in Japan feel that they do not receive sufficient training to undertake this task effectively, although the new approach to interviews, as discussed above, has been piloted where vulnerability has been recognised. We also learned from our aforementioned interactions with Public Prosecutors in Japan that many of the issues presented in this paper were largely unfamiliar to them (i.e. those concerning identification of autism, the way that memories are formed and recalled by those with ASD, the way they may experience the investigative interview, and how certain questioning strategies, such as WAFA, might enhance their ability to provide a detailed account). In addition, we also learned from them that much of the relevant research cited in this paper was not known by the public prosecutors, who were keen to develop their skills in such demanding interviews. As such, the implications of our paper are for it to act as the basis for a training framework that builds on the admirable training developments over the last ten years or so in the areas of interviews with all suspects, with those with mental health conditions and with child interviewees.

### Final thoughts

Current research undertaken across the world is broad in its reach when analysing autism (S. C. Freckelton, 2012; King & Murphy, 2014; Lerner et al., 2012, Maras & Bowler, 2012), with direct reference to criminal justice (Archer & Hurley, 2013; Cheely et al., 2012; Robinson et al., 2012), jurisdictions such as Japan (Fujikawa et al., 2002; Kumagami & Matsuura, 2009; Toichi, 2002) and in regard to the quality of police training (Crane et al., 2016; Hepworth, 2017; National Autistic Society, 2017). What research has emerged tends to focus on suspects and less so on those victims and witnesses with ASD (but see Maras et al., 2020, for promising developments). What is also suggested from the existing research base is that Japan is unlikely to be the only country where professionals identifying and interviewing those with autism need to be better trained. More research is needed to enhance current levels of understanding of ASD across the globe in order to avoid inadequate and inappropriate responses to suspects, witnesses and victims and ultimately to prevent miscarriages of justice.

In developing insights into the challenges facing the Japanese we have highlighted how cultural factors and stigmatisation may also play a part in understanding ASD. However, whilst the education and development of prosecutors in Japan are extensive, it is still doubtful that current legal system prepares prosecutors fully to effectively interview individuals on the ASD scale.

## Ethical standards

## Declaration of conflicts of interest

Dave Walsh has declared no conflicts of interest

Graham Brooks has declared no conflicts of interest

Makiko Naka has declared no conflicts of interest

Gavin Oxburgh has declared no conflicts of interest

Akira Kyo has declared no conflicts of interest

## Ethical approval

This article does not contain any studies with human participants or animals performed by any of the authors.
